# Complete Genome Sequence of a Type Strain of *Mycobacterium abscessus* subsp. *bolletii*, a Member of the *Mycobacterium abscessus* Complex

**DOI:** 10.1128/genomeA.01530-17

**Published:** 2018-02-01

**Authors:** Mitsunori Yoshida, Hanako Fukano, Yuji Miyamoto, Keigo Shibayama, Masato Suzuki, Yoshihiko Hoshino

**Affiliations:** a Department of Mycobacteriology, Leprosy Research Center, National Institute of Infectious Diseases, Tokyo, Japan; b Department of Bacteriology II, National Institute of Infectious Diseases, Tokyo, Japan; c Antimicrobial Resistance Research Center, National Institute of Infectious Diseases, Tokyo, Japan

## Abstract

*Mycobacterium abscessus* subsp. *bolletii* is a rapidly growing mycobacterial organism for which the taxonomy is unclear. Here, we report the complete genome sequence of a *Mycobacterium abscessus* subsp. *bolletii* type strain. This sequence will provide essential information for future taxonomic and comparative genome studies of these mycobacteria.

## GENOME ANNOUNCEMENT

The number of cases of nontuberculosis mycobacteria (NTM) is increasing, especially in developed countries. In our hospital-based survey of pulmonary NTM disease patients in Japan, the number of pulmonary *Mycobacterium abscessus* disease cases increased 5-fold relative to a survey conducted 7 years earlier (1). Moreover, the finding that the multidrug-resistant *M. abscessus* complex (MABC) is transmissible between patients with conditions such as cystic fibrosis provided a radical shift in thinking about MABC acquisition, which was previously thought to be environmental (2–4). Despite advances made in the genomics and documentation of clinical phenotypical differences among MABCs, discrepancies in conventional DNA-DNA hybridization (DDH) results led to debate about MABC taxonomic differentiation (3, 5–9).

Here, we report the complete genome sequence of *M. abscessus* subsp. *bolletii* BD^T^ (=CIP108541^T^). The strain was grown in Middlebrook 7H9 medium, and DNA was extracted using a standard phenol-chloroform method. The genome sequence was determined using PacBio reads (112,992 reads) obtained with the RS II system (Pacific Biosciences, Menlo Park, CA, USA) (10–12). The reads were *de novo* assembled with Canu version 1.5 (13), and the assembled genome was circularized by manually trimming the repeated sequences. Illumina 2 × 300-bp paired-end reads (100,578,382 reads) were obtained with the MiSeq system (Illumina, San Diego, CA, USA) and mapped to the assembly using the Burrows-Wheeler aligner (14) for sequence and assembly error correction with Pilon (15). The DDBJ Fast Annotation and Submission Tool (DFAST) (https://dfast.nig.ac.jp/) was used for annotation (16). Average nucleotide identity (ANI), genome-to-genome distance (GGD), and genomic signature-delta distance (GS-DD) were calculated by JSpeciesWS, Genome-to-Genome Distance Calculator 2.1 (http://ggdc.dsmz.de/ggdc.php), and δ*-differences (http://www.cmbl.uga.edu/software/delta-differences.html), respectively (17–19).

The length of the *M. abscessus* subsp. *bolletii* BD^T^ genome is 5,080,450 bp (64.1% G+C content). Mycobacterium-related ANI was 96.95% to *M. abscessus* subsp. *abscessus* (complete genome of strain ATCC_19977^T^ [20]) and 96.73% to *M. abscessus* subsp. *massiliense* (complete genome of strain JCM_15300^T^ [7]). GGD-estimated DDH values between strains BD^T^ and ATCC_19977^T^, strains ATCC_19977^T^ and JCM_15300, and strains JCM_15300 and BD^T^ were 87.0%, 86.0%, and 88.1%, respectively. The GS-DD was 23 for these 3 comparisons. These values support the proposed taxonomic position of *M. abscessus* subsp. *bolletii* (3). The number of predicted protein-coding sequences in the genome (*n* = 4,976) is nearly equivalent to those of *M. abscessus* subsp. *abscessus* (*n* = 4,920) and *M. abscessus* subsp. *massiliense* (*n* = 4,857) (7, 20). The numbers of rRNA operons (*n* = 3) and tRNA genes (*n* = 49) are similar or equivalent to those of close relatives. Although a draft genome sequence for *Mycobacterium abscessus* subsp. *bolletii* BD^T^ was previously determined (21), the number of predicted genes differed significantly from that obtained here. We also identified a 97.1-kb prophage that was longer than that seen in a previous study (63 kb) (21). The complete genome sequence of *M. abscessus* subsp. *bolletii* BD^T^ represents essential data for future taxonomic and comparative genome studies.

### Accession number(s).

The chromosome sequence was deposited in DDBJ/ENA/GenBank under accession no. AP018436.

## References

[1] Namkoong H, Kurashima A, Morimoto K, Hoshino Y, Hasegawa N, Ato M, Mitarai S 2016 Epidemiology of pulmonary nontuberculous mycobacterial disease, Japan. Emerg Infect Dis 22:1116–1117. doi:10.3201/eid2206.151086. 27191735 PMC4880076

[2] Bryant JM, Grogono DM, Rodriguez-Rincon D, Everall I, Brown KP, Moreno P, Verma D, Hill E, Drijkoningen J, Gilligan P, Esther CR, Noone PG, Giddings O, Bell SC, Thomson R, Wainwright CE, Coulter C, Pandey S, Wood ME, Stockwell RE, Ramsay KA, Sherrard LJ, Kidd TJ, Jabbour N, Johnson GR, Knibbs LD, Morawska L, Sly PD, Jones A, Bilton D, Laurenson I, Ruddy M, Bourke S, Bowler IC, Chapman SJ, Clayton A, Cullen M, Daniels T, Dempsey O, Denton M, Desai M, Drew RJ, Edenborough F, Evans J, Folb J, Humphrey H, Isalska B, Jensen-Fangel S, Jonsson B, Jones AM, 2016 Emergence and spread of a human-transmissible multidrug-resistant nontuberculous mycobacterium. Science 354:751–757. doi:10.1126/science.aaf8156. 27846606 PMC5142603

[3] Hoshino Y, Suzuki K 2015 Differential diagnostic assays for discriminating mycobacteria, especially for nontuberculous mycobacteria: what does the future hold? Future Microbiol 10:205–216. doi:10.2217/fmb.14.120. 25689533

[4] Thomson R, Tolson C, Carter R, Coulter C, Huygens F, Hargreaves M 2013 Isolation of nontuberculous mycobacteria (NTM) from household water and shower aerosols in patients with pulmonary disease caused by NTM. J Clin Microbiol 51:3006–3011. doi:10.1128/JCM.00899-13. 23843489 PMC3754680

[5] Adekambi T, Sassi M, van Ingen J, Drancourt M 2017 Reinstating *Mycobacterium massiliense* and *Mycobacterium bolletii* as species of the *Mycobacterium abscessus* complex. Int J Syst Evol Microbiol 67:2726–2730. doi:10.1099/ijsem.0.002011. 28820087

[6] Leao SC, Tortoli E, Euzéby JP, Garcia MJ 2011 Proposal that *Mycobacterium massiliense* and *Mycobacterium bolletii* be united and reclassified as *Mycobacterium abscessus* subsp. *bolletii* comb. nov., designation of *Mycobacterium abscessus* subsp. *abscessus* subsp. nov. and emended description of *Mycobacterium abscessus*. Int J Syst Evol Microbiol 61:2311–2313. doi:10.1099/ijs.0.023770-0. 21037035

[7] Sekizuka T, Kai M, Nakanaga K, Nakata N, Kazumi Y, Maeda S, Makino M, Hoshino Y, Kuroda M 2014 Complete genome sequence and comparative genomic analysis of *Mycobacterium massiliense* JCM 15300 in the *Mycobacterium abscessus* group reveal a conserved genomic island MmGI-1 related to putative lipid metabolism. PLoS One 9:e114848. doi:10.1371/journal.pone.0114848. 25503461 PMC4263727

[8] Tortoli E, Kohl TA, Brown-Elliott BA, Trovato A, Cardoso-Leão S, Garcia MJ, Vasireddy S, Turenne CY, Griffith DE, Philley JV, Niemann S, Wallace RJ, Cirillo DM 2017 *Mycobacterium abscessus*, a taxonomic puzzle. Int J Syst Evol Microbiol. doi:10.1099/ijsem.0.002457. 29139343

[9] Tortoli E, Kohl TA, Brown-Elliott BA, Trovato A, Leão SC, Garcia MJ, Vasireddy S, Turenne CY, Griffith DE, Philley JV, Baldan R, Campana S, Cariani L, Colombo C, Taccetti G, Teri A, Niemann S, Wallace RJ Jr, Cirillo DM 2016 Emended description of *Mycobacterium abscessus*, *Mycobacterium abscessus* subsp. *abscessus* and *Mycobacterium abscessus* subsp. *bolletii* and designation of *Mycobacterium abscessus* subsp. *massiliense* comb. Nov. Int J Syst Evol Microbiol 66:4471–4479. doi:10.1099/ijsem.0.001376. 27499141

[10] Fukano H, Yoshida M, Katayama Y, Omatsu T, Mizutani T, Kurata O, Wada S, Hoshino Y 2017 Complete genome sequence of *Mycobacterium stephanolepidis*. Genome Announc 5:e00810-17. doi:10.1128/genomeA.00810-17. 28818905 PMC5604778

[11] Yoshida M, Miyamoto Y, Ogura Y, Hayashi T, Hoshino Y 2017 Complete chromosome sequence of a mycolactone-producing mycobacterium, *Mycobacterium pseudoshottsii*. Genome Announc 5:e01363-17. doi:10.1128/genomeA.01363-17. 29192083 PMC5722069

[12] Yoshida M, Nakanaga K, Ogura Y, Toyoda A, Ooka T, Kazumi Y, Mitarai S, Ishii N, Hayashi T, Hoshino Y 2016 Complete genome sequence of *Mycobacterium ulcerans* subsp. *shinshuense*. Genome Announc 4:e01050-16. doi:10.1128/genomeA.01050-16. 27688344 PMC5043562

[13] Koren S, Walenz BP, Berlin K, Miller JR, Bergman NH, Phillippy AM 2017 Canu: scalable and accurate long-read assembly via adaptive k-mer weighting and repeat separation. Genome Res 27:722–736. doi:10.1101/gr.215087.116. 28298431 PMC5411767

[14] Li H, Durbin R 2009 Fast and accurate short read alignment with Burrows-Wheeler transform. Bioinformatics 25:1754–1760. doi:10.1093/bioinformatics/btp324. 19451168 PMC2705234

[15] Walker BJ, Abeel T, Shea T, Priest M, Abouelliel A, Sakthikumar S, Cuomo CA, Zeng Q, Wortman J, Young SK, Earl AM 2014 Pilon: an integrated tool for comprehensive microbial variant detection and genome assembly improvement. PLoS One 9:e112963. doi:10.1371/journal.pone.0112963. 25409509 PMC4237348

[16] Yoshida M, Izumiyama S, Fukano H, Sugiyama K, Suzuki M, Shibayama K, Hoshino Y 2017 Draft genome sequence of Mycobacterium sp. strain shizuoka-1, a novel mycobacterium isolated from groundwater of a bathing facility in Shizuoka, Japan. Genome Announc 5:e01309-17. doi:10.1128/genomeA.01309-17. 29167250 PMC5701475

[17] Richter M, Rosselló-Móra R, Oliver Glöckner F, Peplies J 2016 JSpeciesWS: a web server for prokaryotic species circumscription based on pairwise genome comparison. Bioinformatics 32:929–931. doi:10.1093/bioinformatics/btv681. 26576653 PMC5939971

[18] Auch AF, von Jan M, Klenk HP, Göker M 2010 Digital DNA-DNA hybridization for microbial species delineation by means of genome-to-genome sequence comparison. Stand Genomic Sci 2:117–134. doi:10.4056/sigs.531120. 21304684 PMC3035253

[19] Karlin S, Mrázek J, Campbell AM 1997 Compositional biases of bacterial genomes and evolutionary implications. J Bacteriol 179:3899–3913. doi:10.1128/jb.179.12.3899-3913.1997. 9190805 PMC179198

[20] Ripoll F, Pasek S, Schenowitz C, Dossat C, Barbe V, Rottman M, Macheras E, Heym B, Herrmann JL, Daffé M, Brosch R, Risler JL, Gaillard JL 2009 Non mycobacterial virulence genes in the genome of the emerging pathogen *Mycobacterium abscessus*. PLoS One 4:e5660. doi:10.1371/journal.pone.0005660. 19543527 PMC2694998

[21] Choi GE, Cho YJ, Koh WJ, Chun J, Cho SN, Shin SJ 2012 Draft genome sequence of *Mycobacterium abscessus* subsp. *bolletii* BD^T^. J Bacteriol 194:2756–2757. doi:10.1128/JB.00354-12. 22535937 PMC3347169

